# Geospatial Distribution and Determinants of Undernutrition Among Children Under Five in Oromia Regional State, Ethiopia

**DOI:** 10.1155/jnme/5556781

**Published:** 2025-04-24

**Authors:** Tesfaye Getachew Charkos, Godana Arero, Meyrema Abdo

**Affiliations:** School of Public Health, Adama Hospital Medical College, Adama, Oromia, Ethiopia

**Keywords:** Bayesian, children, Ethiopia, spatial variations, stunting, undernutrition, underweight, wasting

## Abstract

**Background:** Malnutrition is the leading cause of morbidity and mortality among children under five, with significant regional disparities, particularly in Ethiopia, being very high. This study aims to use Geographic Information Systems (GIS) to identify hotspot areas and associated factors for stunting and wasting among children under five in Oromia Regional State, Ethiopia.

**Methods:** A community-based cross-sectional study was conducted. Data were obtained from the 2019 Ethiopian Demographic and Health Survey (EDHS). A total of 653 children under five years old were included in this study. The data were collected using a multistage sampling technique to select the study participants. ArcGis Version 10.7 was used for geospatial analysis. A Bayesian logistic regression model was used to determine the associated factors for undernutrition. A *p* value < 0.05 was considered statistically significant.

**Results:** Overall, the prevalence of stunting and wasting was 36.29% and 4.9%, respectively. In hotspot analysis, both Guji Zone and East Hararge were at high risk of stunting among children under 5 years. Eastern Guji (Gora Dola) and Eastern Hararge (Goro Muti and Meta) areas were at high risk for wasting children under 5 years old. In the adjusted model, being rural residents, mothers who had attended a secondary/above school, children aged 24–35 and 36–47 months, a preceding birth interval > 48 months, using a protected water source, and wealth index were significantly associated with stunting among children under five years. Similarly, mothers aged 25–35 years, rural residents, married women, preceding birth intervals > 48 months, and having more than three children were significantly associated with wasting among children under 5 years.

**Conclusion:** The prevalence of stunting and wasting among children under 5 years remains high in the study setting. These findings suggest that a multifaceted approach addressing education, water access, socioeconomic conditions, and targeted health interventions for high-risk populations is essential to reducing stunting and wasting among children under 5 years old in Oromia Regional State.

## 1. Introduction

Malnutrition is the leading cause of morbidity and mortality in children under five [1]. Childhood malnutrition accounts for 35% of deaths among children under 5 years. Each year, more than 2 million children under the age of five die as a result of undernutrition [2]. In 2015, it was estimated that one-third of children under five in low- and middle-income countries were affected by undernutrition [3]. Moreover, its consequences were related to poor cognitive development, school performance, and productivity [4, 5].

Child undernutrition levels varied significantly across different countries [6]. The variation is particularly pronounced based on regional socioeconomic status, with two-thirds (66%) of all stunted children located in the low- and middle-income countries. Among these, the Asian and African continents account for over half (56%) of all stunted children under 5 years [7]. Similarly, studies indicate that over two-thirds of all wasted children are in Asia, while more than 28% are in Africa. Given the spatial variability in child undernutrition, geographically targeted strategies are essential to address these disparities [8].

The 2019 Ethiopian Demographic and Health Survey (EDHS) revealed significant variation in the prevalence of child undernutrition across regional states in Ethiopia [9]. Specifically, stunting and wasting exhibit notable regional disparities. The highest proportions of stunted children are found in Tigray (48%), Afar (42%), Amhara (42%), and Oromia (35.6%). Conversely, the highest rates of wasting are observed in Somali (21%), Afar (14%), and Gambela (13%), with Oromia having a lower rate of 4.7% [9, 10]. These findings highlight the urgent need for targeted interventions in regions with significant spatial inequalities in childhood undernutrition. By allocating scarce resources to the most affected areas, public health strategies can maximize their impact [11, 12].

While previous studies have highlighted the regional disparities in child undernutrition, there is limited research that specifically focuses on hotspot identification and the associated factors for stunting and wasting at a localized level within the Oromia Regional State. This study fills that critical gap by employing spatial analysis using Geographic Information Systems (GIS) to identify hotspot areas of stunting and wasting among children under five. GIS–based spatial analysis is a powerful tool in public health research, enabling the identification of populations at the highest risk of disease and improving the efficiency of interventions [13–16].

The novelty of this study lies in its focused geographical scope and its use of advanced spatial analysis techniques to uncover localized patterns of undernutrition. Unlike broader national or regional analyses, this research provides granular insights into vulnerable areas within Oromia, thereby offering actionable data for policymakers. By linking spatial data to potential risk factors, this work contributes to a deeper understanding of the drivers of childhood undernutrition and sets the stage for tailored, high-impact interventions.

## 2. Methodology

### 2.1. Study Area and Design

**Figure 1:**
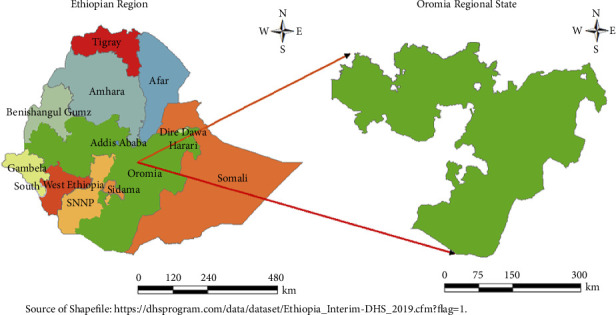
Map of the study area of Oromia National Regional State and Ethiopian regions.

A community-based cross-sectional study was conducted in the Oromia National Regional State (Figure 1). According to the 2011 census, Oromia has a total population of approximately 37.27 million, with 15% under the age of five and 54% under 18 years old. The fertility rate in Oromia is higher than the national average, with a total fertility rate of 5.4 for women aged 15–49, compared to the national rate of 4.6 [17].

### 2.2. Inclusion and Exclusion Criteria

**Figure 2:**
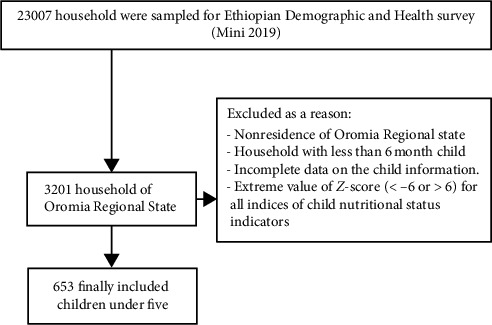
The process of participant inclusion and exclusion criteria from EDHS 2019.

The source population for this study comprised all households with children under five residing in the Oromia Regional State during the study period. Children aged 6–59 months who had lived in the selected households for more than six months were included. Exclusion criteria are detailed in Figure 2. The study analyzed 35 enumeration areas (EAs) from the EDHS 2019, with all participants from these EAs recruited. Samples were selected using a stratified, two-stage cluster sampling design.

### 2.3. Study Variables and Measurements

The dependent variable in this study was the undernutrition status of children under five, including stunting and wasting. Independent variables encompassed sociodemographic factors such as the age of the child and mother, child's sex, family size, household wealth index, parental education level, occupation, religion, maternal marital status, and age at first marriage. Child-specific characteristics included age in months, preceding birth interval, initiation and exclusivity of breastfeeding, and current breastfeeding status. Community-level factors included place of residence, type of latrine facility, and water source.

### 2.4. Operational Definition

• Child stunting: It is an anthropometric index that reflects the long-term cumulative effects of inadequate nutrition and health. It is defined as low height-for-age at < −2 SD of the median value of the NCHS/WHO international growth reference [18].
• Child wasting: Wasting refers to low weight-for-height at < −2 SD of the median value of the NCHS/WHO international growth reference [18].
• Child underweight: An anthropometric index of weight-for-age represents body mass relative to age, defined as low weight-for-age at < −2 SD of the median value of the NCHS/WHO international growth reference [18]. A child can be underweight for his/her age because he or she is stunted, wasted, or both. Weight-for-age is an overall indicator of a population's nutritional health [19].

### 2.5. Data Analysis

Descriptive statistics were presented as mean (standard deviation) for normally distributed continuous variables and as frequency (percentage) for categorical variables. Spatial analysis was performed using ArcGIS Version 10.7.1. The global Moran's I statistic was applied to assess the spatial autocorrelation of undernutrition among children under five in the study area [19–21]. The statistical significance of clustering was determined using Getis-Ord Gi^∗^ statistics, based on *Z*-scores and *p* values [20, 22]. If the *Z*-score falls outside the expected range, the observed spatial pattern is likely too unusual to be attributed to random chance, resulting in a small *p* value. A high Gi^∗^ value identifies a “hotspot” (high-risk area) for undernutrition among children, while a low Gi^∗^ value indicates a “cold spot” (low-risk area) [20, 23, 24]. Spatial interpolation was used to predict values in unsampled areas based on data from sampled locations (ordinary Kriging spatial) [25].

A Bayesian logistic regression model was employed for the analysis in this study, as this approach does not rely on *p* values to assess the statistical significance of the relationships between dependent and independent variables [26, 27]. *p* values can provide misleading evidence due to their dependence on sample size. In Bayesian modeling, prior information must be specified, so we assumed that each variable's coefficient followed a normal distribution with a mean of zero and a variance of 10,000. The model estimation involved 10,000 iterations, with the first 5000 used as the burn-in period.

The association between associated factors and the nutritional status of children under five was reported using adjusted odds ratios (ORs) with 95% credible intervals (CrI). The goodness of fit was assessed using the deviance information criterion (DIC). Multicollinearity among variables was checked with the variance inflation factor (VIF), using a threshold of less than 5. All analyses were performed with STATA Version 17.0 (StataCorp, College Station, Texas, United States of America).

## 3. Results

### 3.1. Characteristics of the Study Participants

A total of 653 households with children under five were recruited from the 2019 EDHS. The median age of the mothers was 28 years (interquartile range [IQR]: 24–34). The majority of these mothers were (619 [94.8%]) married and (572 [87.6%]) resided in rural areas. One-third of the households relied on unprotected sources of drinking water. Over half of the mothers were Muslim (53.6%) and 349 (53%) were illiterate. Among the participants, 315 (48.2%) of the households had two under-five children, and 324 (49.6%) households were classified as poor (Table 1).

### 3.2. Maternal and Child Care Practices of Under-Five Children

Of the total under-five children, 330 (50.5%) were male. The median age of the children under five was 28 months (IQR: 15–43 months). A majority of mothers (382 [58%]) delivered at home, and 184 (40.3%) had more than four ANC visits during pregnancy. Approximately half of the mothers (261 [49.9%]) had a preceding birth interval between 24 and 47 months. Three hundred forty-nine mothers (53.5%) practiced exclusive breastfeeding, and during data collection, 427 (71.4%) mothers were in the breastfeeding stage. Regarding immunizations, 230 (59.3%) of the children under five received the BCG vaccine, and of these, 136 (35.1%) were vaccinated for measles (Table 2).

### 3.3. Prevalence of Undernutrition (Stunting, Wasting, and Underweight)

**Figure 3:**
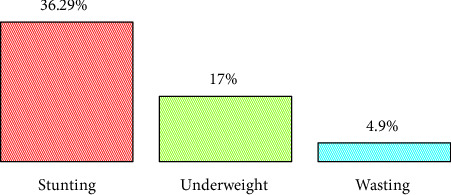
The prevalence of stunting, underweight, and wasting, respectively, in Oromia Regional State–based EDHS 2019 data.

This study revealed that the prevalence of stunting, underweight, and wasting was 36.29% (95% CI: 32.55%–39.99%), 17% (95% CI: 14.12%–19.88%), and 4.9% (95% CI: 3.2%–6.6%), respectively, among children under five in Oromia Regional State based on data from EDHS 2019 (Figure 3).

### 3.4. A Spatial Autocorrelation Analysis

**Figure 4:**
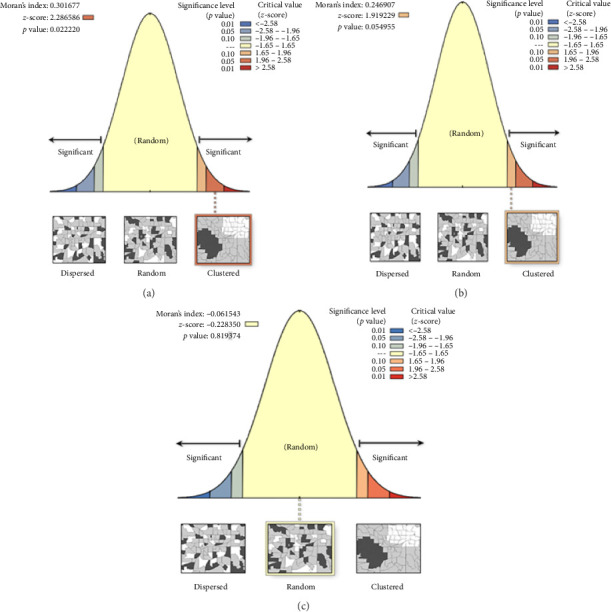
Spatial autocorrelation of stunting (a), wasting (b), and underweight (c) among children under five across the Oromia Regional States.

The spatial autocorrelation analysis indicates a statistically significant positive clustering of stunting and wasting among under-five children. The results showed that stunting and wasting had statistically significant positive spatial autocorrelation (MI = 0.30, *p*=0.022; MI = 0.25, *p*=0.045, respectively). Similarly, the *Z*-scores for stunting and wasting were 2.28 and 1.92, respectively, further indicating a clustered pattern (Figure 4(a) and 4(b)). However, there was no statistically significant spatial autocorrelation for underweight children under five in the Oromia Regional State (MI = −0.06, *p*=0.82). Similarly, the *Z*-score of −0.228 indicates that the clustering pattern of underweight is not significant (Figure 4(c)).

#### 3.4.1. Hotspot Analysis

**Figure 5:**
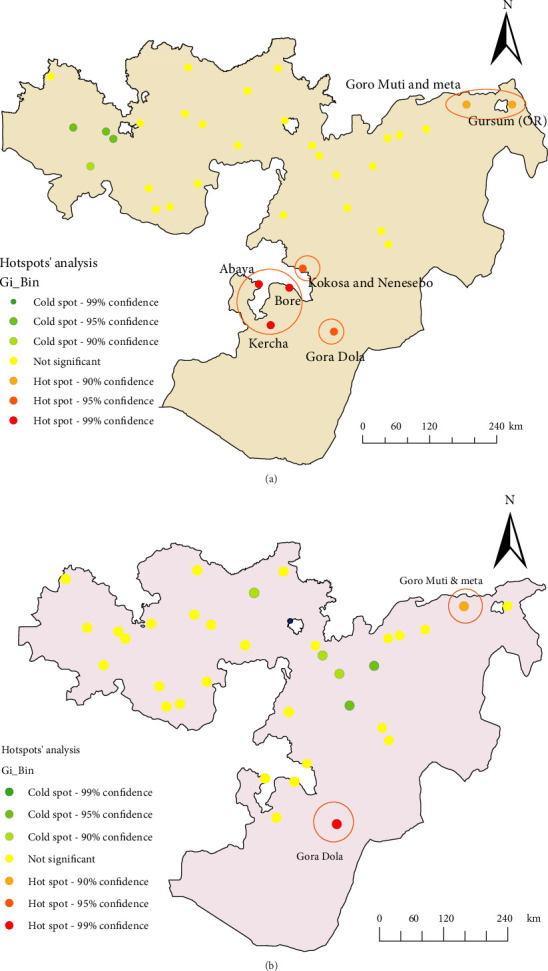
Hotspot analysis of children under five for stunting and wasting in Oromia, Ethiopia: Based on EDHS 2019 data.

The hotspot analysis reveals that the Eastern and Western Guji zones, as well as the East Hararge areas (Goro Muti, Meta, and Gursum), are at high risk for stunting among children under five (Figure 5(a)). Similarly, the Eastern Guji area (Gora Dola) and the Eastern Hararge areas (Goro Muti and Meta) were identified as high-risk zones for wasting among children under five (Figure 5(b)). Consistently, the EDHS 2019 data revealed that stunting and wasting among children under five were concentrated in specific areas. The hotspot map highlights the geographic distribution of stunting and wasting hotspots in Oromia, Ethiopia, based on 2019 EDHS data. It shows clusters of high prevalence for stunting (low height-for-age) and wasting (low weight-for-height), with regions identified as high-risk and those with lower prevalence. This visualization is essential for targeting nutritional interventions and allocating resources effectively.

#### 3.4.2. Spatial Interpolation

**Figure 6:**
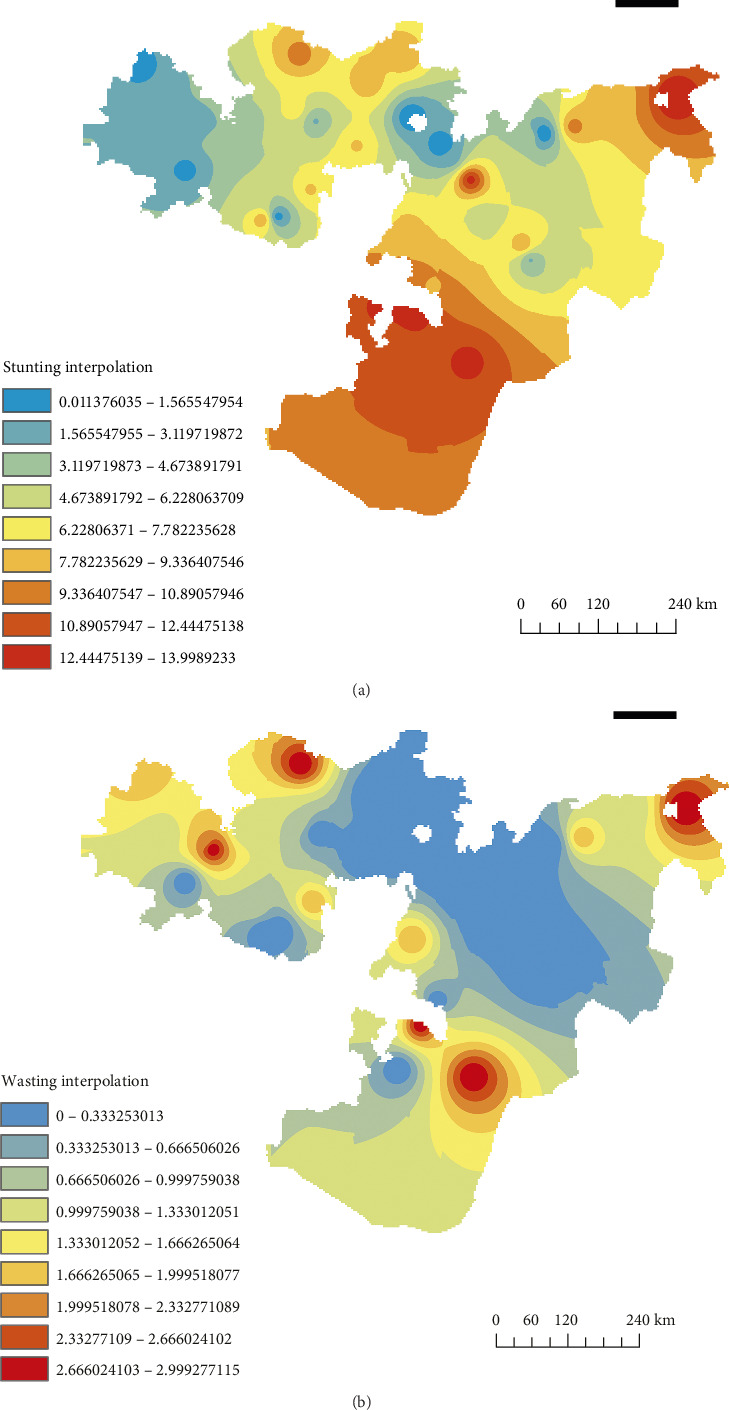
Spatiotemporal interpolation of stunting and wasting among children under five in the Oromia Regional State, Ethiopia.

Figure 6(a) shows the interpolation of high-risk areas for stunting based on available data. The predictions indicate that the southern, northeastern, and northwestern, were at high risk for stunting among children. Similarly, Figure 6(b) highlights high-risk areas for wasting, including the surrounding Guji Zone, and the northeastern, western, and central regions of Oromia.

### 3.5. Factors Associated With Stunting Under-Five

In multivariable Bayesian logistic regression analysis, the odds of stunting among children under five whose parents were rural residents were 3.21 (OR: 3.21; 95% CrI: 1.56–5.93) times more likely than those whose parents were urban residents. Regarding mother's education, the odds of stunting among children under five whose mothers have attended a secondary/above school were 0.23 (OR: 0.23; 95% CrI: 0.09–0.46) times less likely than illiterate mothers. In this study, those children between 24 and 35 (OR: 3.67; 95% CrI: 2.17–5.77) and 36 and 47 (OR: 3.27; 95% CrI: 1.57–6.06) months of age were more likely to face stunting than children in 6–11 months. Having a preceding birth interval greater than 48 months, 53% (OR: 0.47; 95% CrI: 0.23–0.87), reduces the odds of stunting among children under five than those mothers who have a less than 24-month birth interval in the Oromia Regional State. A family who used a protected water source for drinking, 44% (OR: 0.56; 95% CrI: 0.32–0.85), reduced the odds of stunting among children under five compared to those households who used unprotected water. Children from rich families were, 39% (OR: 0.61; 95% CrI: 0.40–0.84), less likely to face stunting compared to poor households (Table 3).

#### 3.5.1. Factors Associated With Wasting Under-Five

The odds of wasting among children under five whose mothers aged 25–35 years were (OR: 7.52; 95% CrI: 1.81–23.59) more likely than counterparts. Similarly, rural residents were (OR: 15.83; 95% CrI: 1.69–52.57) more than those whose parents were urban residents. Being married, women were less likely to waste children under five in the Oromia Regional State (OR: 0.11; 95% CrI: 0.02 and 0.33). Having a preceding birth interval greater than 48 months, 53% (OR: 5.33; 95% CrI: 1.23–15.25), increases the odds of wasting among children under five than counterparts. A family who had more than three children was (OR: 18.79; 95% CrI: 2.44–62.60) more likely to waste among children under five compared to their counterparts (Table 3).

## 4. Discussion

This study aimed to determine the geospatial distribution and determinants of undernutrition among children under five years old in the Oromia Regional State, based on the EDHS 2019 data. In this community-based cross-sectional study, we observed a prevalence of 36.29% for stunting, 4.9% for wasting, and 17% for underweight, respectively. Several studies reported consistent findings with ours [14, 28, 29]. In those studies, the prevalence of stunting was reported to range between 35.4% and 37.7%. Similarly, our study found that stunting among children under 5 years old remains alarmingly high, indicating a persistently severe public health issue in our study setting. In addition, our findings align with those from research conducted in the Southern Nations, Nationalities, and Peoples' (SNNP) Regional State (35.4%) [29], further highlighting the widespread nature of this problem. In a similar context, the prevalence of stunting was reported to be as high as 34.4% in the Somali region [30], 39% in Kenya [31], 37.7% in Padanpur [32], and 35.4% in the Bench Maji, Ethiopia [33], which is comparable to our findings. However, some studies in Ethiopia have reported a higher prevalence of stunted children under five compared to our current findings [34, 35]. This underscores the ongoing challenge of addressing stunting in these regions and the need for targeted public health interventions.

This study observed a prevalence of 4.9% for wasting among children in the Oromia Regional State. Since 2000, the prevalence of both wasting and being underweight has been declining in the Oromia Regional State, from 11.6% in 2000 to 4.9% in 2019, and from 38.1% in 2000 to 16.1% in 2019, respectively [10, 36]. Over the past two decades, there has been a significant reduction in the prevalence of wasting and underweight among children in the Oromia Regional State. This improvement is likely the result of the cumulative efforts of various stakeholders, including global, national, and local interventions. These efforts have contributed to enhanced access to formal education, improved health services, better information and communication systems, and increased household food security. Collectively, these factors have played a crucial role in addressing the underlying causes of malnutrition in the region [37, 38].

In the adjusted model, several factors were found to be significantly associated with stunting among children in our study setting. These factors include the place of residence of the household, the child's age, the mother's level of education, the birth interval, the source of drinking water, and the household wealth index. Each of these variables played a significant role in influencing the likelihood of stunting, highlighting the complex interplay of socioeconomic and environmental factors in child health outcomes.

We found that children residing in rural areas faced a higher risk of stunting compared to those from urban areas. This finding was consistent with several previous studies that have reported a greater prevalence of undernutrition among preschool children in rural settings [15, 39, 40]. In addition, our results were in agreement with a study using data from the 2016 EDHS, which indicated that children from rural areas had higher odds of stunting compared to those from urban areas [41]. The significant disparity between rural and urban areas can be attributed to various socioeconomic factors, including differences in maternal education, household wealth, and food security. In rural areas, lower levels of maternal education often correlate with limited knowledge about nutrition and child care, which can contribute to higher rates of stunting. In addition, lower household wealth in rural settings can restrict access to quality food and healthcare services. Food insecurity, which is more prevalent in rural areas, further aggravates the risk of undernutrition among children. These factors collectively contribute to the pronounced gap in stunting rates between rural and urban populations [15]. On the other hand, severe undernutrition in rural areas is often worsened by delays or refusals to seek medical care due to financial constraints and logistical challenges. Economic hardships, long distances to healthcare facilities, and lack of transportation make it difficult for families to access timely medical intervention and nutritional support, intensifying the problem.

This study found that the odds of stunting among children under five were significantly lower for those whose mothers had attended secondary school or higher, compared to those whose mothers were illiterate. These findings are supported by previous studies [42, 43]. Educated mothers are more likely to access modern healthcare, possess better health knowledge, and engage in healthier reproductive behaviors compared to those with no formal education [42, 43]. Similarly, a cross-sectional study found that children from families with no literacy or informal education faced a 4.2 times higher risk of stunting compared to those from families with university or college education [44]. The evidence highlights the importance of educational attainment for mothers as a key factor in improving child health outcomes and reducing undernutrition.

This study found that children aged 24–47 months were more likely to experience stunting compared to those under 11 months. This indicates that the risk of stunting increases with age. However, the data for children aged 47–59 months was not statistically significant due to a smaller sample size in this age group. Consistent with our findings, previous studies have also shown a direct relationship between stunting and age [45, 46]. In addition, a birth interval of more than 48 months significantly reduces the odds of stunting and increases wasting in children under five compared to a birth interval of less than 24 months. This is likely because, after a 24-month interval, mothers are better able to provide focused care and resources for the newborn. Longer intervals allow more time for recovery and resource allocation, improving child-rearing practices and ensuring that older children receive adequate nutrition and care.

Among environmental determinants, the source of drinking water was found to be a significant predictor of stunting in our study. Families using a protected water source had a 44% lower risk of stunting in children under five compared to those using unprotected water. These results are consistent with similar findings from studies in Ethiopia [47]. These findings align with similar studies in Ethiopia, which suggest that stunting can be reduced by promoting safe drinking water, handwashing practices, and access to clean water [47]. The justification for these findings is that protected water sources lower the risk of waterborne diseases and contamination, which helps prevent malnutrition and stunting. In addition, promoting handwashing and ensuring access to clean water improve child health by preventing infections and enhancing sanitation, leading to better nutritional outcomes and reduced stunting risk.

This study found that children from wealthier households were 39% less likely to experience stunting compared to those from poorer households. This aligns with previous research [48] indicating that stunting is associated with low socioeconomic conditions, poor maternal health, inadequate nutrition, inappropriate feeding practices, and frequent early-life hospital admissions. Wealthier families typically have better access to nutrition and medical care, which contributes to the lower risk of stunting among their children.

In this study, children whose mothers were aged 25–35 years had a significantly higher risk of wasting compared to those with younger mothers. This finding is consistent with previous cross-sectional studies in Ethiopia [49, 50]. Older mothers may have more children and might struggle to provide adequate nutrition and medical care, increasing the likelihood of wasting among their children. This may be attributed to the greater workload older mothers face, which can make it challenging to meet their children's nutritional and health needs, thereby increasing the risk of wasting.

We found that family size was significantly associated with wasting in children under five. Households with more than three children were more likely to experience higher rates of wasting compared to those with fewer children. A larger family size can strain resources, making it more difficult to provide adequate nutrition and care for all children. Households with more than three children may face increased competition for limited resources, which can contribute to higher rates of wasting. This is consistent with previous studies [51, 52], which highlight the challenges larger families face in meeting the nutritional needs of their children.

## 5. Conclusion

In our study, the prevalence of stunting and wasting among children under five was 36.29% and 4.9%, respectively. Hotspot analysis identified high-risk areas for stunting in the Guji Zone and East Hararge, while Eastern Guji (Gora Dola) and Eastern Hararge (Goro Muti and Meta) were at high risk for wasting.

The adjusted model revealed that stunting was significantly associated with being a rural resident, having mothers with secondary or higher education, children aged 24–35 and 36–47 months, a preceding birth interval greater than 48 months, access to protected water sources, and higher wealth index. For wasting, significant associations were found with mothers aged 25–35 years, rural residence, marital status, a preceding birth interval greater than 48 months, and families with more than three children. Undernutrition remains a persistent problem in the Oromia Regional State. Therefore, we recommend focused interventions to address these challenges effectively.

## Figures and Tables

**Table 1:** Sociodemographic characteristics of respondents of the Oromia Regional State (EDHS 2019).

Characteristics	Category	Frequency	Percent
Mother's age (in years)	< 25	276	42.3
	25–35	288	44.1
	> 35	89	13.6

Marital status of mothers	Married	619	94.8
	Others^a^	34	5.2

Place of residence	Urban	81	12.4
	Rural	572	87.6

Source of drinking water	Unprotected	410	62.8
	Protected	243	37.2

Mother's religion	Muslim	346	53.0
	Protestant	210	32.2
	Others^b^	97	14.8

Mother's education level	Illiterate	349	53.5
	Primary	258	39.5
	Secondary and above	46	7.0

Number of under-five children	1	226	34.6
	2	315	48.2
	> 3	112	17.2

Wealth index	Poor	324	49.6
	Middle	144	22.1
	Rich	185	28.3

Presence of latrine	Yes	444	68.0
	No	209	32.0

^a^Single, divorced, and widowed.

^b^Orthodox, Wakefata, and Catholic.

**Table 2:** Maternal and child care practices of children under five in Oromia based on the EDHS 2019.

Characteristics	Category	Frequency	Percent
Sex of child's	Male	330	50.5
	Female	323	49.5

Child age in months	6–11	128	19.6
	12–23	124	19.0
	24–35	136	20.8
	36–47	146	22.4
	48–59	119	18.2

Place of delivery	Home	382	58.5
	Health facility	271	41.5

ANC follow-up	No visit	130	28.5
	1–3 visits	143	31.3
	> 4 visits	184	40.3

Preceding birth interval	< 24 months	144	27.5
	24–47 months	261	49.9
	> 48 months	118	22.6

Initiation of breastfeeding	Immediately	364	82.4
	Later	78	17.6

Exclusive breastfeeding	Yes	349	53.5
	No	304	46.5

Currently breastfeeding	Yes	427	71.4
	No	171	28.6

BCG vaccinated	Yes	230	59.3
	No	158	40.7

Measles vaccinated	Yes	136	35.1
	No	252	64.9

**Table 3:** Bayesian logistic analysis on the factors associated with undernutrition (stunting and wasting) among children under five in Oromia, Ethiopia, based on EDHS 2019.

Variables		Stunting status	Wasting status
		AOR (95% CrI)	AOR (95% CrI)
Age of mothers	25–35 years	1.50 (0.98, 2.41)	**7.52 (1.81, 23.59)**
	≥ 35 years vs	0.83 (0.52, 1.33)	2.30 (0.37, 8.88)
	≤ 25 years	1	1

Place of residence	Rural	**3.21 (1.56, 5.93)**	**15.83 (1.69, 52.57)**
	Urban	1	1

Mothers educational level	Primary	0.94 (0.52, 1.61)	0.80 (0.21, 2.02)
	Secondary/above	**0.23 (0.09, 0.46)**	1.02 (0.21, 2.76)
	Illiterate	1	1

Mothers' marital status	Married	1.77 (0.76, 4.67)	**0.11 (0.02, 0.33)**
	Others^∗^	1	1

Sex of child	Female	0.65 (0.39, 1.10)	0.60 (0.22, 1.44)
	Male	1	1

The child's age in months	12–23 months	0.72 (0.47, 1.05)	2.98 (0.81, 8.42)
	24–35 months	**3.67 (2.17, 5.77)**	0.97 (0.16, 3.05)
	36–47 months	**3.27 (1.57, 6.06)**	0.57 (0.02, 2.70)
	6–11 months	1	1

Place of delivery	Health facility	0.74 (0.46, 1.12)	2.47 (0.52, 7.55)
	Home	1	1

ANC follow-up	1–3 visits	**2.12 (1.21, 3.82)**	1.28 (0.43, 2.90)
	4 plus or more	**2.61 (1.54, 3.81)**	0.64 (0.13, 1.80)
	No visits	1	1

Preceding birth interval	24–47 months	0.67 (0.41, 1.05)	2.13 (0.43, 8.26)
	> 48 months	**0.47 (0.23, 0.87)**	**5.33 (1.23, 15.25)**
	< 24 months	1	1

Water source	Protected vs	**0.56 (0.32, 0.85)**	0.59 (0.15, 1.50)
	Unprotected	1	1

Toilet type	Yes	1.31 (0 95, 1.76)	2.55 (0.62, 7.12)
	No	1	1

Number of U5C in households	Two	0.96 (0.59, 1.48)	4.93 (0.98, 17.68)
	≥ 3 children	1.29 (0 0.58, 2.64)	**18.79 (2.44, 62.60)**
	One or fewer child	1	1

Exclusive breastfeeding	Yes vs	1.44 (0.68, 2.60)	0.38 (0.08, 1.15)
	No	1	1

The wealth index of the households	Middle	2.60 (0.76, 1.65)	1.14 (0.34, 3.56)
	Rich	**0.61 (0.40, 0.84)**	1.18 (0.05, 3.52)
	Poor	1	1

*Note:* Bold indicates statistically significant.

Abbreviation: CrI = credible interval.

^∗^Single, divorced, widowed, and separated.

## Data Availability

The quantitative data used to support the findings of this study are available from the corresponding author upon request.

## Nomenclature

CrI: Credible interval
CI: Confidence interval
EAs: Enumeration areas
EDHS: Ethiopian Demographic and Health Survey
NCHS: National Center for Health Statistics
OR: Odds ratio
SD: Standard deviation
SNNPR: South Nation Nationality People Region
WHO: World Health Organization
